# A retrospective cohort pilot study to evaluate a triage tool for use in a pandemic

**DOI:** 10.1186/cc8146

**Published:** 2009-10-29

**Authors:** Michael D Christian, Cindy Hamielec, Neil M Lazar, Randy S Wax, Lauren Griffith, Margaret S Herridge, David Lee, Deborah J Cook

**Affiliations:** 1 Department of National Defence -- Canadian Forces, Mount Sinai Hospital Toronto/University Health Network, University of Toronto, 600 University Avenue, Toronto, ON, Canada, M5G 1X5; 2 Hamilton Health Sciences Corporation, McMaster University, 237 Barton Street East, Hamilton, ON, Canada, L8L 2X2; 3 University Health Network, University of Toronto, 200 Elizabeth Street, Toronto, ON, Canada, M5G 2C4; 4 Mount Sinai Hospital Toronto, University of Toronto, 600 University Avenue, Toronto, ON, Canada, M5G 1X5; 5 McMaster University, 1280 Main Street West, Hamilton, ON, Canada, L8S4L8; 6 University of Toronto, 600 University Avenue, Toronto, ON, Canada, M5G 1X5; 7 St Josephs Health Care Centre, McMaster University, 50 Charlton Avenue East, Hamilton, ON, Canada, L8N 4A6

## Abstract

**Introduction:**

The objective of this pilot study was to assess the usability of the draft Ontario triage protocol, to estimate its potential impact on patient outcomes, and ability to increase resource availability based on a retrospective cohort of critically ill patients cared for during a non-pandemic period.

**Methods:**

Triage officers applied the protocol prospectively to 2 retrospective cohorts of patients admitted to 2 academic medical/surgical ICUs during an 8 week period of peak occupancy. Each patient was assigned a treatment priority (red -- 'highest', yellow -- 'intermediate', green -- 'discharge to ward', or blue/black -- 'expectant') by the triage officers at 3 separate time points (at the time of admission to the ICU, 48, and 120 hours post admission).

**Results:**

Overall, triage officers were either confident or very confident in 68.4% of their scores; arbitration was required in 54.9% of cases. Application of the triage protocol would potentially decrease the number of required ventilator days by 49.3% (568 days) and decrease the total ICU days by 52.6% (895 days). On the triage protocol at ICU admission the survival rate in the red (93.7%) and yellow (62.5%) categories were significantly higher then that of the blue category (24.6%) with associated *P *values of < 0.0001 and 0.0003 respectively. Further, the survival rate of the red group was significantly higher than the overall survival rate of 70.9% observed in the cohort (*P *< 0.0001). At 48 and 120 hours, survival rates in the blue group increased but remained lower then the red or yellow groups.

**Conclusions:**

Refinement of the triage protocol and implementation is required prior to future study, including improved training of triage officers, and protocol modification to minimize the exclusion from critical care of patients who may in fact benefit. However, our results suggest that the triage protocol can help to direct resources to patients who are most likely to benefit, and help to decrease the demands on critical care resources, thereby making available more resources to treat other critically ill patients.

## Introduction

On 11 June, 2009 the World Health Organization acknowledged that the world was facing a pandemic caused by a novel strain of H1N1 influenza [1]. Although to date the overall prevalence of severe H1N1 illness has been low, experiences in Mexico [2,3] and Manitoba [4] have increased concern that scarcities of critical care resources, such as mechanical ventilators [5,6], will occur if a large second wave strikes during the fall in the Northern Hemisphere. Surge response strategies [7-11] will partially mitigate the surge impact, but may be inadequate to fully address health care demands. When faced with scarce resources, the principles of biomedical ethics and international law dictate that triage protocols be used to guide resource allocation [12-14].

In 2006, an expert panel convened by the Ontario Health Plan for an Influenza Pandemic Steering Committee developed a draft critical care triage protocol [15]. The purpose of the protocol is to provide guidance for making triage decisions if critical care services are overwhelmed [5,6,16,17].

The objective of this pilot study was to assess the usability of the draft triage protocol, to estimate its potential impact on patient outcomes, and its potential to increase resource availability based on a cohort of critically ill patients cared for during a non-pandemic period.

## Materials and methods

We identified two retrospective cohorts of consecutive patients admitted to two academic medical/surgical intensive care units (ICUs) during an eight-week period of peak occupancy. ICU 'A' is a 16-bed general medical-surgical unit with 800 to 850 annual admissions serving a 472-bed tertiary care hospital and a 220-bed cancer hospital. ICU 'B' is a 23-bed medical-surgical-trauma-neurosurgical ICU with approximately 1200 admissions per year serving a 972-bed tertiary care hospital. Research ethics board approval was obtained at each of the participating institutions. The requirement for informed consent was waived by the research ethics board. Trained research assistants abstracted data from patient charts to create the case summary. An intensivist member of the study team at each site was available to answer clinical questions and review difficult cases. All case report forms were reviewed by the principal investigator, MDC, who requested additional data or site investigator chart review as necessary to clarify missing or unclear information. Data were entered into an Excel spreadsheet (Microsoft Excel 2003, Microsoft Corporation. USA).

**Figure 1:**
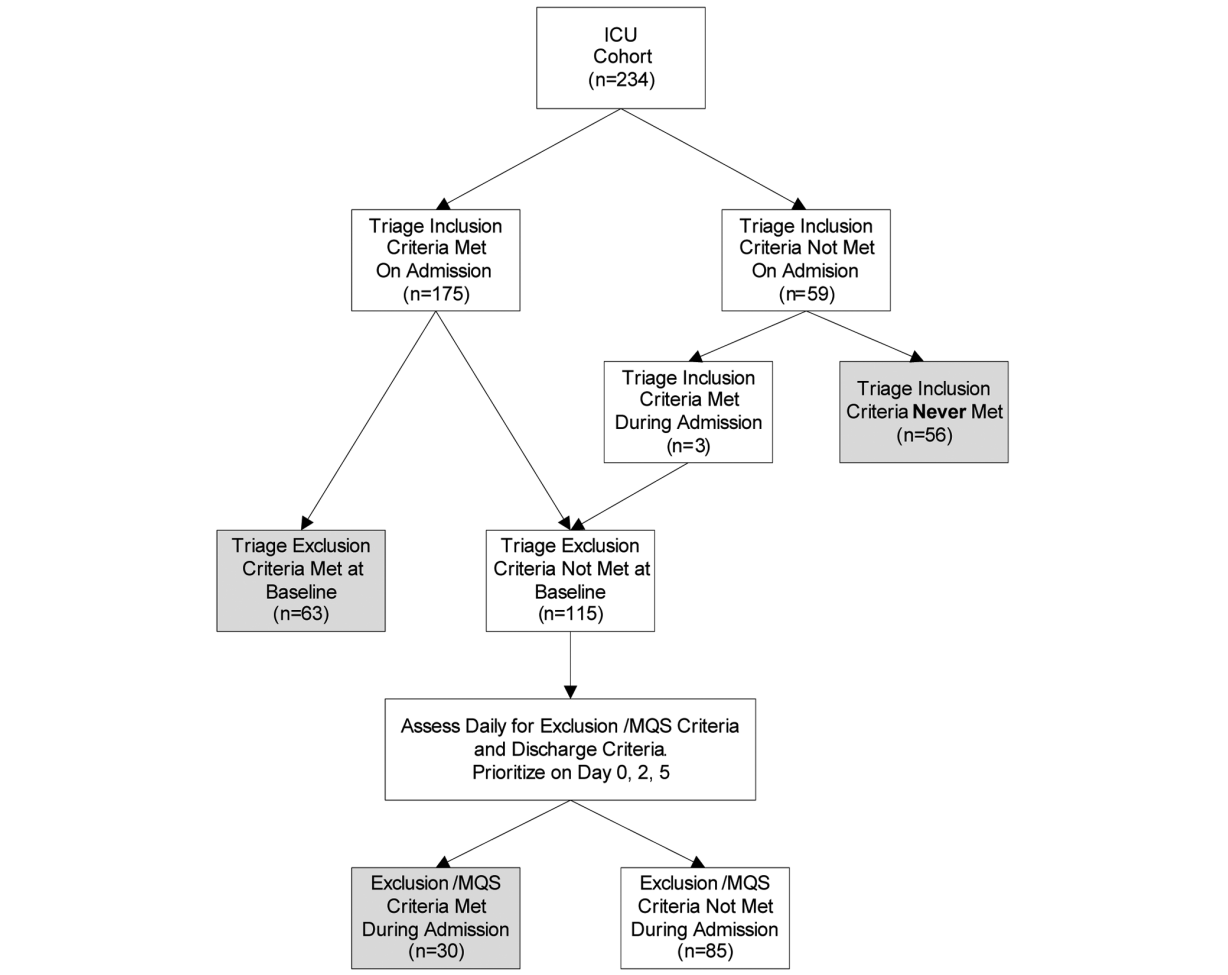
Triage process flow. ICU = intensive care unit; MQS = minimum qualifications for survival.

An overview of the triage process is presented in Figure 1. Research assistants identified all patients admitted to these two participating ICUs from the ICU admission logs. Patients were enrolled into the study if they met the inclusion criteria for ICU admission defined in the triage protocol [see Additional data file 1] [15]. Patients were then screened to see if they met any of the protocol's initial exclusion criteria or 'minimum qualifications for survival' (MQS) [see Additional data file 1] [15]. Any patients who did not fulfill the triage protocol's initial exclusion criteria were summarized and presented to two of four intensivists who served as 'triage officers' to apply the triage protocol. Three of the four officers were involved in drafting the Ontario triage protocol. All triage officers received one hour of training on how to apply the protocol for this study (three in a single session and the fourth was one-on-one with the principal investigator due to scheduling difficulties). The training focus was triaging sample cases and calibration of the protocol application. Triage officers were instructed to imagine that they were actually conducting triage during a pandemic where demand for critical care services exceeded the available capacity. For study purposes, they were instructed that following the triage protocol was to be considered the 'standard of care' during the pandemic period. Deviations from the protocol would hold the same potential risks and consequences as do deviations from the standard of care during non-pandemic situations.

Patients at each of the two sites were scored independently by two of the triage officers. The triage officers did not review patients from the institution in which they practice, did not discuss these cases with one another during the triage process, and had no prior knowledge of the patients or their outcomes. Patient profiles were presented electronically in PDF format to the triage officers. The profiles included the patient's demographic data, admission diagnosis and limited past medical history. Daily triage reports were presented to the triage officers with the sequential organ failure assessment (SOFA) score component data and a calculated SOFA score, one day at a time, for each of the first five ICU days. The triage officers were instructed to make and record their determination of the patient's treatment priority before advancing to the next day's triage report, although they were allowed to move back in the PDF to review the patient profile and prior day's SOFA scores. Thus, at the time of their triage assessments, the triage officers were blinded to future status, and patient outcome.

Each patient was assigned a treatment priority (red = 'highest', yellow = intermediate', green = 'discharge to ward', or blue/black = 'expectant') by the triage officers at three separate time points (at the time of admission to the ICU, and 48, and 120 hours after admission). In addition, on day 1, 3 and 4 of their ICU admission, patients were assessed by the triage officer for exclusion criteria. Disagreements were resolved by a third intensivist (the principal investigator) scoring the case, in the same manner as described above, blinded to the decisions of the other triage officers and patient outcomes.

Protocol endpoints due to triage protocol exclusion criteria events or reaching discharge criteria beyond day 5 were assessed by a research assistant in conjunction with the principal investigator. We recorded the date and vital status of the patient at discharge from the ICU and hospital, or at day 90 following enrollment if the patient was still in the hospital.

Usability of the draft triage protocol was assessed based on: intensivist rating of confidence in their assignment of triage priority as reported in a brief questionnaire using five-point Likert-style scales assessing completeness of data, relevance of information provided, ability to develop a clear clinical understanding of the case and overall confidence in decision; requirement for third intensivist assessment; and chance-corrected (kappa) in assignment of triage priority. Based on the usability criteria, the triage protocol was considered successful in its current format if overall confidence was rated as confident or very confident in at least 90% of cases and disagreements requiring a third assessment in less then 5% of cases.

We assessed the impact of the triage protocol on resource availability by calculating the difference in days between when a patient achieved an endpoint in the triage protocol, exclusion criteria or prioritized as either blue 'expectant' or green 'dischargeable', compared with the actual date of extubation and discharge from the ICU. This allowed calculation of the number of ICU and ventilator days made available under the triage protocol.

Patient status was recorded at discharge from ICU, discharge from hospital or day 90 following enrollment if the patient remained admitted to hospital. Patients were categorized as deceased or alive at discharge from ICU and hospital, or if still admitted to hospital patients were categorized as: 'requiring ongoing life support', 'undergoing active medical care', or 'awaiting transfer to chronic care/rehabilitation'. Patients who were discharged from hospital alive had their destination of discharge categorized during chart abstraction as: 'home', 'chronic care', 'rehabilitation' or 'transfer to another acute care facility'.

### Statistical analysis

We present descriptive data using mean and standard deviation for continuous variables, or median and interquartile ranges if data were skewed, and proportions and 95% confidence intervals for dichotomous variables. We compared continuous variables using unpaired t-tests (means) or Wilcoxon Rank-Sum (medians), and dichotomous variables using a chi-squared test or a Fisher's exact test if any expected cell frequencies were less than five. All tests were two-sided and *P *< 0.05 was considered statistically significant. We used Cohen's kappa (κ) with 95% confidence intervals to calculate chance-corrected agreement between triage officers. Based on the usability criteria the triage protocol would be considered successful in its current format if overall confidence is rated as confident or very confident in at least 90% of cases and disagreements requiring third assessment in less then 5% of cases. Missing data for the calculation of the SOFA score were assumed to be normal if the parameter was never collected by the clinicians caring for the patient, or the last reported value was used if the parameter had previously been measured but was missing at the time in question.

## Results

A total of 234 patients were included in the cohort (Figure 1 and Table 1), of which 178 (76.1%) met the triage inclusion criteria and would have been admitted to ICU during a pandemic based on the protocol. Of the overall cohort, 39.7% at some point met either the triage exclusion criteria or MQS and thus would have been managed expectantly (triaged blue). The number of patients who met triage inclusion or exclusion criteria was similar between hospitals. The mean age of the cohort was 59.8 years old and was similar between hospitals. A lower percentage of patients in hospital B were women, most likely because it is a trauma center. Overall, 69.2% of patients were mechanically ventilated, while 5.1% were never ventilated but were hypotensive and required vasopressor support. Overall, ICU survival was 76.9% and hospital survival was 70.9%.

**Table 1:** Cohort description.

	Hospital A	Hospital B	Total	*P*
N	113	121	234	
Age, mean (sd)	60.4 (18.0)	59.3 (19.0)	59.8 (18.5)	
Age range	21-96	19-92	19-96	
Gender; n (% female)	58 (51.3)	42 (34.7)	100 (42.7)	0.10
Ventilated; n (%)	72 (63.7)	90 (74.4)	162 (69.2)	0.08
Hypotension; n (%)	11 (9.7)	1 (0.8)	12 (5.1)	0.002
Met triage inclusion	82 (72.6)	96 (79.3)	178 (76.1)	0.23
Met triage exclusion/MQS	45 (39.8)	48 (39.7)	93 (39.7)	0.98
ICU survival; n (%)	84 (74.3)	96 (79.3)	180 (76.9)	0.36
Hospital survival; n (%)	77 (68.1)	89 (73.6)	166 (70.9)	0.36
Hosp LOS; median (IQR)	9 (3, 20)	12 (5, 23)	10.5 (4, 21)	0.06
Ventilator days for patients meeting triage inclusion criteria; median (IQR); (n)	2 (1, 7) (n = 82)	2.5 (1, 7) (n = 96)	2 (1, 7) (n = 178)	0.99
Ventilator days on protocol; median (IQR)	1 (0, 2)	1 (0, 5)	1 (0, 3)	0.24
ICU LOS for patients meeting triage inclusion criteria; median (IQR); (n)	3 (2, 9) (n = 82)	5.5 (3, 11) (n = 96)	5 (2, 10) (n = 178)	0.07
ICU LOS on protocol; median (IQR)	2 (0, 3)	3 (0, 7.5)	2 (0, 5)	0.05

ICU = intensive care unit; IQR = inter quartile range; LOS = length of stay; MQS = minimum qualifications for survival; n = number; sd = standard deviation.

The primary outcome measure was the usability of the triage protocol as measured by triage officer confidence in their assignment of priority, requirement for arbitration and agreement with each other. Overall, triage officers were either confident or very confident in 68.4% of their scores and arbitration was required in 54.9% of cases. The agreement between raters (Table 2) was highest on admission (kappa 89.2 (77.9, 100) and 73.7 (55.6, 91.7) for hospitals A and B, respectively. Agreement at 48 hours and 120 hours were substantially lower (48 hours: kappa 60.7 (41.2, 80.2) and 31.5 (16.1, 46.8); 120 hours: kappa 42.4 (28.0, 57.0) and 0 (-17.2, 16.2) for hospitals A and B). The latter analyses were based on fewer patients because only patients who were not triaged blue and were still in the ICU were triaged at these subsequent time points.

**Table 2:** Usability of protocol

	Triage officer				
	1	2	3	4	Overall
Mean rating of completeness of data (95% CI)	4.5 (4.3, 4.8)	4.5 (4.3, 4.7)	4.0 (4.0, 4.0)	3.9 (3.8, 4.1)	4.2 (4.1, 4.4)
Mean rating of relevance of information (95% CI)	4.6 (4.3, 4.8)	4.5 (4.3, 4.7)	4.0 (4.0, 4.0)	4.0 (3.9, 4.1)	4.3 (4.1, 4.4)
Mean rating of ability to develop clear clinical picture (95% CI)	4.5 (4.3, 4.7)	4.5 (4.3, 4.7)	3.4 (3.1, 3.6)	3.9 (3.8, 4.1)	4.1 (3.9, 4.2)
Mean rating of confidence in triage decisions (95% CI)	4.3 (4.0, 4.6)	4.1 (3.9, 4.4)	3.1 (2.9, 3.4)	3.9 (3.7, 4.1)	3.9 (3.7, 4.0)
Percentage rating of confident or very confident in their triage decisions (95% CI)	76.9	72.2	46.0	76.3	68.4
Percentage of cases requiring arbitration	27.8%		79.7%		54.9%
Agreement with arbitrator N (%)	10 (66.7)	5 (33.3)	2 (4.3)	45 (95.7)	n/a

Ratings based on five-point Likert-style scale.

CI = confidence interval; n = number; n/a = not available.

Secondary outcome measures of this pilot study included an assessment of the impact of the triage protocol on the availability of critical care resources and measurement of patient outcomes. Total ventilator and ICU days on and off protocol are reported in Table 3. Application of the triage protocol would potentially decrease the number of ventilator days required by 49.3% (568 days) and a decrease of 52.6% (895 days) in the total ICU days. Table 4 presents outcomes based on triage category at admission, 48 hours and 120 hours. On the triage protocol at ICU admission the survival rate in the red (93.7%) and yellow (62.5%) categories were significantly higher then that of the blue category (24.6%) with associated *P *value of less than 0.0001 and 0.0003, respectively. Further, the survival rate of the red group was significantly higher than the overall survival rate of 70.9% observed in the cohort (*P *< 0.0001). At 48 hours the survival rates in the red (90.5%) and yellow (94.1%) categories remain significantly higher than that of the blue category (45.4%) with *P *values of 0.002 and 0.001; however, beyond 48 hours the number of patients remaining in the ICU had decreased substantially, limiting inferences from these analyses.

**Table 3:** Cumulative resource utilization

	Actual	On protocol
Ventilator days	1152	584
Intensive care unit length of stay	1701	806

**Table 4:** Survival rate by triage category

	Triage category			
	Red	Yellow	Green	Blue
	**Admission**			
**N**	79	32	0	65
**Survived**^† ^n (%)	74 (93.7)	20 (62.5)		16 (24.6)
% Difference c/w Blue (95% CI)	69.1^1^ (57.3, 80.8)	37.9^2^ (18.1, 57.7)	-	-
	**48 hours**			
**n**	21	17	29	22
**Survived**^† ^n (%)	19 (90.5)	16 (94.1)	29 (100)	10 (45.4)
% Difference c/w Blue (95% CI)	45.0^3^ (20.7, 69.3)	48.7^4^ (25.0, 72.3)	-	-
	**120 hours**			
**n**	6	10	14	7
**Survived**^† ^n (%)	6 (100)	9 (90.0)	13 (92.9)	5 (71.4)
% Difference c/w Blue (95% CI)	28.6 (-4.9, 62.0)	18.6 (-19.7, 56.9)	-	-

^† ^survived to hospital discharge, CI = confidence interval; c/w = compared with.

^1^*P *< 0.0001, ^2^*P *= 0.0003, ^3^*P *= 0.002, ^4^*P *= 0.001.

To examine the outcomes of those patients who would have been triaged to the blue category yet survived in the observation cohort, the outcomes and criteria for those ever triaged to blue category (Table 5) are compared with outcomes for those never triaged to blue category (Table 6). Of the patients triaged to blue, 32.3% survived to hospital discharge. Almost half of the survivors triaged to the blue category had been triaged blue for failing to sufficiently improve their SOFA score at either 48 or 120 hours. The most common exclusion criterion triggering a triage to category blue in those patients who survived to hospital discharge was the presence of metastatic cancer.

**Table 5:** Analysis of patients who were ever triaged blue or met exclusion criteria

	Survived			Expired		
	Exclusion criteria/MQS	triaged blue	Total	Exclusion criteria/MQS	triaged blue	Total
**N**	13	17	30	43	20	63
90-day status*				N/A	N/A	N/A
N (%)	0	1 (5.9)	1 (3.3)	N/A	N/A	N/A
Life support (%)				N/A	N/A	N/A
Medical care (%)		1	1	N/A	N/A	N/A
Awaiting cc/rehab				N/A	N/A	N/A
**Discharged**				N/A	N/A	N/A
N (%)				N/A	N/A	N/A
Home	6 (46.1)	7 (23.3)	13 (43.3)	N/A	N/A	N/A
Chronic care	4 (30.8)	0	4 (13.3)	N/A	N/A	N/A
Acute care	3 (23.1)	5 (29.4)	8 (26.7)	N/A	N/A	N/A
Rehab	0	5 (29.4)	5 (16.7)	N/A	N/A	N/A
**Time of endpoint**						
Admission	13 (100)	3 (17.7)	16 (53.3)	40 (93.0)	9 (45.0)	49 (77.8)
48 hours		10 (58.8)	10 (33.3)	1 (2.3)	11 (55.0)	12 (19.1)
120 hours		4 (23.5)	4 (13.3)	0	0	0
other		N/A	0	2 (4.7)	0	2 (3.2)
**Blue triage** **SOFA criteria**						
Admission > 11	N/A	3		N/A	9	
48 hour > 11	N/A	1		N/A	6	
48 hour 8-11 & no change	N/A	9		N/A	4	
120 hour > 11	N/A	0		N/A	1	
120 hour < 8 & no change	N/A	4		N/A	0	
**Exclusion criteria**						
Severe trauma	0	N/A		4	N/A	
Severe burn	0	N/A		0	N/A	
Cardiac arrest	1	N/A		12	N/A	
Severe cognitive impairment	0	N/A		0	N/A	
Advanced neuromuscular disease	0	N/A		0	N/A	
Metastatic malignancy	5	N/A		6	N/A	
Advanced & irreversible immunocompromise	0	N/A		0	N/A	
Severe & irreversible neurologic event/condition	1	N/A		12	N/A	
End-stage heart failure	1	N/A		1	N/A	
End-stage lung failure	0	N/A		3	N/A	
End-stage liver failure	1	N/A		0	N/A	
Age > 85	3	N/A		10	N/A	
> 6 u PRBC within 24 hours	1	N/A		1	N/A	
Elective palliative surgery	1	N/A		0	N/A	
SOFA > 11 non-triage days	0	N/A		2	N/A	

* if still admitted to hospital

cc = chronic care; MQS = minimum qualifications for survival; N/A = not available; PRBC = packed red blood cells; rehab = rehabilitation; SOFA = sequential organ failure assessment.

**Table 6:** Analysis of survivors who met inclusion criteria but never triaged blue or excluded

	Never triage blue/excluded
N (%)	81
90-day status*	
N (%)	2 (2.5)
Life support (%)	
Medical care (%)	1
Awaiting cc/rehab	1
**Discharged**	
N (%)	80^1^
Home	41 (51.3)
Chronic care	6 (7.5)
Acute care	20 (25.0)
Rehab	12 (15.2)

^1^Missing data for one patient

cc = chronic care; rehab = rehabilitation.

Following the triage exercise, triage officers were polled to assess if they believed they were the 'type' of person who could make triage decisions; if they would volunteer to be a triage officer in a pandemic; and their view regarding the training required to be a triage officer. All except triage officer 3 said they believed they were the type of person who could make triage decisions and would volunteer in a pandemic. All stated that specific comprehensive training for intensivists to be triage officers is required.

## Discussion

This study provides insight into the use of triage protocols during pandemic periods, and informs the design for a larger multi-center prospective study to evaluate the Ontario triage protocol. Our results highlight the need to develop a selection process for triage officers and to provide comprehensive training for triage officers. Many cases required arbitration due to disagreement between triage officers 3 and 4. The arbitrator ruled in agreement with triage officer 4 on 95.7% of the decisions.

The overall degree of confidence in triage decisions fell below the *a priori *target of greater than 90% of decisions being rated as either confident or very confident. Additionally, the rate of arbitration was high, although primarily the result of decisions by one triage officer. A lack of consistency between triage officers threatens the equity of the process. The clinical information provided about patients was much less then intensivists would typically receive in clinical practice, and triage officers reported that the data were insufficient to establish a clear clinical picture in 20.5% of cases (data not shown). Thus, more specific clinical information will have to be provided in a future triaging study, which may be available during prospective application of the protocol, but increasing dependency on information can limit the usability of the protocol. Other possible reasons for lower than anticipated confidence among triage officers and high rate of arbitration is inexperience with the protocol, inadequate training, and the lack of prior studies exploring the impact of triage protocols such as this. Future qualitative research could better illuminate factors that contribute to lack of confidence in triage decision-making using a protocol such as the one studied.

Although not specifically designed or powered to evaluate the impacts of the triage protocol on patient outcome, this study provides some insight into its performance. The primary goal of a tertiary triage protocol is to direct the limited available resources to those who are most likely to benefit from them. On this point, the protocol appears to serve its function with those triaged to the highest priority for ICU care (red) having survival rates significantly higher than the rate in the observation cohort and markedly higher than those triaged blue. The alternative to the use of a protocol would be leaving individual physicians to make allocation decisions on their own. Prior research suggests that such ICU physicians' ability to predict patient outcome without the aid of a decision support tool, such as a triage protocol, is poorer than we have observed in this study [18]. Additionally, failure to use a standard triage protocol to guide decisions regarding the allocation of scarce resources is less efficient and ethically less desirable [19-21].

A second goal of tertiary triage is to make more critical care resources available. On this point, the protocol shows promise. Through the application of the protocol's inclusion criteria, exclusion criteria, MQS and discharge criteria, the demand for ventilators could be decreased by 49.3% and for ICU admission by 52.6% compared with standard practices. As an illustrative example, based upon the 568 days of ventilation made available through the use of the protocol, using rates from the first wave of H1N1 in Canada assuming an average of 10 days of ventilation and an 89% survival rate, 50 lives could potentially be saved by the resources made available.

Ethical frameworks suggest that restrictions placed on the allocation of scarce resources must be proportionate to the expected and observed shortfalls [22]. Thus, it is particularly important to minimize the number of people who are triaged blue but may possibly survive under normal circumstances. Evaluating this outcome is somewhat difficult in this study given that patients were receiving what is essentially optimum care in tertiary ICUs, whereas during a pandemic, triage would be instituted only when emergency mass critical care is being used which requires significantly modified standards of care. Thus, one would expect the mortality rate in the sickest of patients (those excluded or triaged blue) would be higher than seen in this observation cohort. Further, survival in this study was defined as status at hospital discharge, whereas the triage protocol was designed to direct resources to patients with greater than 50% two-year survival. Many of the patients who survived to hospital discharge but would have been triaged blue or excluded, such as those with metastatic malignancies, are unlikely to meet the higher bar of greater than 50% two-year survival. Patients are often repatriated to the facilities from which they were transferred once they no longer require services only available at tertiary centres. As we were unable to collect information from the referring facilities in this study, some of these patients may not have survived to discharge from the referring facility.

This study is limited in that it is a pilot study of a relatively small cohort of critically ill patients from two Ontario hospitals. Although clinical data were presented to triage officers prospectively, data were less than would typically be available in practice, and some data were missing. Our results highlight the need for a central triage committee, comprehensive training, and data management infrastructure so the committee can monitor triage outcomes during a pandemic, adjusting the protocol to correct for either over-triage or under-triage [15,23-25]. Further, the exclusion criteria and prioritization criteria should be incorporated into triage decisions on a graduated basis depending upon the anticipated shortfall in resource supply. Additionally, the protocol exclusion, MQS and prioritization criteria require further study and modification to minimize the potential for denying resources to those who may benefit from them.

## Conclusions

We evaluated the Ontario critical care triage protocol in this pilot study, which generated insights about future triage practices. Although further research is needed, our results suggest that the triage protocol can help to direct resources to patients who are most likely to benefit from them, and help to decrease the demands on critical care resources, thereby making available more resources to treat other critically ill patients. We also documented the need for more comprehensive patient summaries when such decisions are being made, careful triage officer selection, improved triage officer training, and infrastructure to allow timely tracking and analysis of the consequences of triage protocols.

## Key messages

• Attention must be paid to the appropriate training and selection of triage officers in to improve confidence in their decisions and agreement between triage officers.

• Application of the triage protocol can potentially free up significant critical care resources, which may be re-directed towards managing critically ill patients during a pandemic or disaster.

• The current protocol is able to identify patients who are most likely to survive and allow resources to be targeted to this group. However, the protocol does require further investigation and modification to minimize the number of patients who would potentially be excluded critical care in a pandemic but whom may survive if they were to receive critical care.

## Abbreviations

ICU: intensive care unit; MQS: minimum qualifications for survival; SOFA: sequential organ failure assessment.

## Competing interests

The authors declare that they have no competing interests.

## Authors' contributions

MC and DC designed the study and secured funding. MC, CH, NL, RW, MH, and DL participated in data collection. LG, MC and DC conducted the data analysis. MC and DC produced the original manuscript draft. All authors participated in editing and reviewing the manuscript. All authors read and approved the final manuscript.

## Supplementary Material

Additional file 1 Word file that provides the detailed inclusion and exclusion criteria that form part of the triage protocol. These are in greater detail then were published in the original article detailing the triage protocol, which is referenced in this paper. Click here for file

## References

[B1] Zarocostas J World Health Organization declares A (H1N1) influenza pandemic BMJ 2009 338 b2425 10.1136/bmj.b2425 19525308

[B2] Perez-Padilla R de la Rosa-Zamboni D Ponce de Leon S Hernandez M Quiñones-Falconi F Bautista E Ramirez-Venegas A Rojas-Serrano J Ormsby CE Corrales A Higuera A Mondragon E Cordova-Villalobos JA Pneumonia and Respiratory Failure from Swine-Origin Influenza A (H1N1) in Mexico N Engl J Med 2009 361 680 689 10.1056/NEJMoa0904252 19564631

[B3] Novel Swine-Origin IA Emergence of a Novel Swine-Origin Influenza A (H1N1) Virus in Humans N Engl J Med 2009 360 2605 2615 10.1056/NEJMoa0903810 19423869

[B4] Kermode-Scott B Canada has world's highest rate of confirmed cases of A/H1N1, with Aboriginal people hardest hit BMJ 2009 339 b2746 10.1136/bmj.b2746 19581332

[B5] Christian MD Devereaux AV Dichter JR Geiling JA Rubinson L Definitive care for the critically ill during a disaster: current capabilities and limitations: from a Task Force for Mass Critical Care summit meeting, January 26-27, 2007, Chicago, IL Chest 2008 133 8S 17S 10.1378/chest.07-2707 18460503 PMC7094433

[B6] Osterholm MT Preparing for the next pandemic N Engl J Med 2005 352 1839 1842 10.1056/NEJMp058068 15872196

[B7] Hick JL Hanfling D Burstein JL DeAtley C Barbisch D Bogdan GM Cantrill S Health care facility and community strategies for patient care surge capacity Ann Emerg Med 2004 44 253 261 10.1016/j.annemergmed.2004.04.011 15332068 PMC7118880

[B8] Rubinson L Nuzzo JB Talmor DS O'Toole T Kramer BR Inglesby TV Augmentation of hospital critical care capacity after bioterrorist attacks or epidemics: recommendations of the Working Group on Emergency Mass Critical Care Crit Care Med 2005 33 2393 2403 10.1097/01.CCM.0000173411.06574.D5 16215397

[B9] Rubinson L Hick JL Curtis JR Branson RD Burns S Christian MD Devereaux AV Dichter JR Talmor D Erstad B Medina J Geiling JA Task Force for Mass Critical Care Definitive care for the critically ill during a disaster: medical resources for surge capacity: from a Task Force for Mass Critical Care summit meeting, January 26-27, 2007, Chicago, IL Chest 2008 133 32S 50S 10.1378/chest.07-2691 18460505 PMC7094478

[B10] Rubinson L Hick JL Hanfling DG Devereaux AV Dichter JR Christian MD Talmor D Medina J Curtis JR Geiling JA Task Force for Mass Critical Care Definitive care for the critically ill during a disaster: a framework for optimizing critical care surge capacity: from a Task Force for Mass Critical Care summit meeting, January 26-27, 2007, Chicago, IL Chest 2008 133 18S 31S 10.1378/chest.07-2690 18460504 PMC7094361

[B11] Rubinson L O'Toole T Critical care during epidemics Critical Care 2005 9 311 313 16137366 10.1186/cc3533 PMC1269436

[B12] Baskett PJ Ethics in disaster medicine Prehospital Disaster Med 1994 9 4 5 10155487 10.1017/s1049023x00040747

[B13] Domres B Koch M Manger A Becker HD Ethics and triage Prehospital Disaster Med 2001 16 53 58 11367943 10.1017/s1049023x00025590

[B14] Vollmar LC Military Medical Ethics Military Medicine In War: The Geneva Conventions Today 2005 Chapter 23

[B15] Christian MD Hawryluck L Wax RS Cook T Lazar NM Herridge MS Muller MP Gowans DR Fortier W Burkle FM Development of a triage protocol for critical care during an influenza pandemic CMAJ 2006 175 1377 1381 17116904 10.1503/cmaj.060911 PMC1635763

[B16] Ministry of Health and Long-Term Care Ontario Health Plan for an Influenza Pandemic http://www.health.gov.on.ca/english/providers/program/emu/pan_flu/pan_flu_plan.html

[B17] Public Health Agency of Canada Canadian Pandemic Influenza Plan 2004

[B18] Rodriguez RM Wang NE Pearl RG Prediction of poor outcome of intensive care unit patients admitted from the emergency department Crit Care Med 1997 25 1801 1806 10.1097/00003246-199711000-00016 9366761

[B19] Challen K Bentley A Bright J Walter D Clinical review: mass casualty triage--pandemic influenza and critical care Crit Care 2007 11 212 17490495 10.1186/cc5732 PMC2206465

[B20] Kuschner WG Pollard JB Ezeji-Okoye SC Ethical triage and scarce resource allocation during public health emergencies: tenets and procedures Hosp Top 2007 85 16 25 10.3200/HTPS.85.3.16-25 17711810

[B21] O'Laughlin DT Hick JL Ethical issues in resource triage Respir Care 2008 53 190 197 18218150

[B22] University of Toronto Joint Centre for Bioethics Pandemic Influenza Working Group Stand On Guard For Thee: Ethical considerations in preparedness planning for pandemic influenza http://www.jointcentreforbioethics.ca/people/documents/upshur_stand_guard.pdf

[B23] Devereaux A Christian MD Dichter JR Geiling JA Rubinson L Summary of suggestions from the Task Force for Mass Critical Care summit, January 26-27, 2007 Chest 2008 133 1S 7S 10.1378/chest.08-0649 18460502 PMC7094306

[B24] Devereaux AV Dichter JR Christian MD Dubler NN Sandrock CE Hick JL Powell T Geiling JA Amundson DE Baudendistel TE Braner DA Klein MA Berkowitz KA Curtis JR Rubinson L Task Force for Mass Critical Care Definitive care for the critically ill during a disaster: a framework for allocation of scarce resources in mass critical care: from a Task Force for Mass Critical Care summit meeting, January 26-27, 2007, Chicago, IL Chest 2008 133 51S 66S 10.1378/chest.07-2693 18460506

[B25] Frykberg ER Medical management of disasters and mass casualties from terrorist bombings: how can we cope? J Trauma 2002 53 201 212 10.1097/00005373-200208000-00001 12169923

