# Supplementary material for: A retrospective cohort pilot study to evaluate a triage tool for use in a pandemic
Source: Crit Care. 2009 Oct 29;13(5):R170. doi: 10.1186/cc8146 (PMC2784402; doi:10.1186/cc8146)
Supplement: Additional file 1 — Word file that provides the detailed inclusion and exclusion criteria that form part of the triage protocol. These are in greater detail then were published in the original article detailing the triage protocol, which is referenced in this paper. [file cc8146-S1.doc]

Appendix

Triage Protocol Inclusion and Exclusion Criteria

Inclusion criteria:

1. Requirement for invasive ventilatory support:

- Refractory Hypoxemia (SpO2 < 90% on non-rebreather mask/ FiO2 > 0.85)
- Respiratory Acidosis with pH < 7.2
- Clinical evidence of impending respiratory failure
- Inability to protect or maintain airway (altered level of consciousness, significant secretions or other airway issue)

1. Hypotension:

- Hypotension (SBP < 90 or relative hypotension) with clinical evidence of shock (altered level of consciousness, decreased urine output, or other end organ failure) refractory to volume resuscitation requiring vasopressor/inotrope support.

Exclusion criteria:

The patient is excluded from admission or transfer to critical

care if *any* of the following is present:

A. Severe trauma

***- a Trauma Injury Severity Score (TRISS) with predicted mortality of >80%, see calculator at http://www.sfar.org/scores2/triss2.html***

B. Severe burns of patient with any 2 of the following:

• Age > 60 yr

• > 40% of total body surface area affected

• Inhalation injury

C. Cardiac arrest

• Unwitnessed cardiac arrest

• Witnessed cardiac arrest, not responsive to electrical

therapy (defibrillation or pacing)

• Recurrent cardiac arrest

***- a second cardiac arrest less than 72h following return of spontaneous circulation and stabilization following successful electrical therapy for initial malignant arrythmia***

D. Severe baseline cognitive impairment

***- a patient who is unable to perform activities of daily living (AODLs) independently due to cognitive impairment OR is institutionalized due to cognitive impairment (i.e. a person who is post devastating traumatic brain injury, advanced Alzheimer's, etc)***

E. Advanced untreatable neuromuscular disease

F. Metastatic malignant disease

G. Advanced and irreversible immunocompromise

***-Most commonly this would be due to AIDS where there are NO antiviral treatment options available or rarely one of the congenital immunocompromise conditions***

H. Severe and irreversible neurologic event or condition

I. End-stage organ failure meeting the following criteria:

*Heart*

• NYHA class III or IV heart failure

- ***Class I: patients with no limitation of activities; they suffer no symptoms from ordinary activities.***
- ***Class II: patients with slight, mild limitation of activity; they are comfortable with rest or with mild exertion.***
- ***Class III: patients with marked limitation of activity; they are comfortable only at rest.***
- ***Class IV: patients who should be at complete rest, confined to bed or chair; any physical activity brings on discomfort and symptoms occur at rest\***

*Lungs*

• COPD with FEV1 < 25% predicted, baseline

PaO2 < 55 mm Hg, or secondary pulmonary

hypertension

• Cystic fibrosis with post bronchodilator FEV1 < 30% or

baseline PaO2 < 55 mm Hg

• Pulmonary fibrosis with VC or TLC < 60% predicted,

baseline PaO2 < 55 mm Hg, or secondary pulmonary

hypertension

• Primary pulmonary hypertension with NYHA class III

or IV heart failure, right atrial pressure > 10 mm Hg,

or mean pulmonary arterial pressure > 50 mm Hg

*Liver*

• Child–Pugh score ≥ 7

- 1. Total Serum Bilirubin
     1. Bilirubin <2 mg/dl: 1 point
     2. Bilirubin 2-3 mg/dl: 2 points
     3. Bilirubin >3 mg/dl: 3 points
  2. Serum Albumin
     1. Albumin >3.5 g/dl: 1 point
     2. Albumin 2.8 to 3.5 g/dl: 2 point
     3. Albumin <2.8 g/dl: 3 point
  3. INR
     1. INR <1.70: 1 point
     2. INR 1.71 to 2.20: 2 point
     3. INR >2.20: 3 point
  4. Ascites
     1. No Ascites: 1 point
     2. Ascites controlled medically: 2 point
     3. Ascites poorly controlled: 3 point
  5. Encephalopathy
     1. No Encephalopathy: 1 point
     2. Encephalopathy controlled medically: 2 point
     3. Encephalopathy poorly controlled: 3 point

J. Age > 85 yr

K. Elective palliative surgery

***-Surgery that is intended for symptomatic relief in a patient with an otherwise terminal condition (ie/ cancer) OR for which the average 2 year survival is less than 50%.***
